# *In Vitro* Study and Comparison of Caecal Methanogenesis and Fermentation Pattern in the Brown Hare (*Lepus europaeus*) and Domestic Rabbit (*Oryctolagus cuniculus*)

**DOI:** 10.1371/journal.pone.0117117

**Published:** 2015-01-28

**Authors:** Dorota Miśta, Bożena Króliczewska, Milan Marounek, Ewa Pecka, Wojciech Zawadzki, Józef Nicpoń

**Affiliations:** 1 Department of Biostructure and Animal Physiology, Wroclaw University of Environmental and Life Sciences, Wrocław, Poland; 2 Institute of Animal Science, Prague, Czech Republic; 3 Department of Internal Medicine and Clinic of Diseases of Horses, Dogs and Cats, Wroclaw University of Environmental and Life Sciences, Wrocław, Poland; University of Lleida, SPAIN

## Abstract

The brown hare and the domestic rabbit are mid-sized herbivorous mammals and hindgut fermenters, though their digestive physiologies differ in some traits. The objective of this study was to estimate and compare the caecal microbial activity in hares and rabbits via an analysis of the following end-products of *in vitro* caecal fermentation: methane, total gas production, short chain fatty acids and ammonia concentration. Hare caecal methanogenesis occurred at a much lower level (0.25 mmol/kg for samples incubated without substrate and 0.22 mmol/kg for samples incubated with substrate) than that of the rabbit (15.49 and 11.73 mmol/kg, respectively) (*P*<0.001). The impact of the substrate’s presence on caecal methanogenesis was not significant, though its presence increased the total gas production during fermentation (*P*<0.001). Hare caecal microflora produced a lower short chain fatty acids concentration than did rabbit microorganisms (*P*<0.05). In unincubated hare samples, the short chain fatty acids concentration was 28.4 mmol/kg, whereas in unincubated rabbit samples, the short chain fatty acids concentration was 51.8 mmol/kg. The caecal fermentation pattern of the hare was characterised by higher propionate and isobutyrate molar proportions compared with those observed in rabbit caecum (*P*<0.01). No significant changes in the ammonia concentration in either rabbit or hare caecum were found. The results obtained indicate some differences in the activity of the microbial populations colonising the hare and rabbit caecum, particularly in regards to methanogenic Archaea.

## Introduction

The brown hare *(Lepus europaeus)* and the domestic rabbit *(Oryctolagus cuniculus)* belong to the leporid family (Leporidae), which comprises mammals that are hindgut fermenters and practitioners of caecotrophy. The leporids produce two types of faeces: soft and hard. During the formation of hard faeces, fine particles and water-soluble substances, together with microorganisms, are brought back to the caecum via antiperistaltic movements and retrograde flow occurring in the proximal colon, and the large particles that form the hard faeces pass to the distal colon [1, 2]. These large particles consist of poorly digestible or less nutritious ingesta particles. In contrast, soft faeces originate directly from the caecal content and contain high concentrations of protein, minerals and vitamins [2, 3, 4]. Rabbits form soft faeces as a cluster surrounded by a mucilaginous membrane, which are consumed and then continue to ferment in the stomach for several hours. In contrast, hares produce amorphous soft faeces that lack a membrane and become barely distinguishable from other materials in the stomach once ingested [4, 5, 6, 7].

The digestive physiology of hares and rabbits presents both similarities and differences. Hares and rabbits have different digestive strategies to cope with low quality of forage. Hares maximize the processing of the food by increasing intake rate and decreasing digesta retention time. Rabbits maximize digestion of the food by increasing the mean digesta retention time [7]. Moreover, Stott [8] found that the caecum of the brown hare, like its other digestive organs, had a relatively smaller size compared with that of the rabbit and that the gut passage rate was significantly faster in hares than in rabbits. Both species are poor digesters of fibre, but the ability to digest hemicelluloses is greater in the rabbit [8].

The caecum is the biggest compartment for fibre degradation and fermentation processes for both hares and rabbits. The caecum is rich in microorganisms, primarily anaerobic bacteria, that metabolise the nutrients leaving the small intestine and produce short chain fatty acids (SCFA), gases (methane, carbon dioxide, hydrogen), ammonia and compounds that are incorporated into the microbial cells [9]. SCFA are important as a source of energy for basic metabolic processes such as growth and lipogenesis, and they play a key role in maintaining health and controlling the intestinal environment [10, 11].

Methanogenesis is a consequence of a series of metabolic interactions among various groups of microorganisms. In addition to bacteria, which are a dominant microbial population in the rabbit caecum, methanogenic Archaea make an essential contribution (up to 22% of total microbial RNA in rabbit caecum), and *Methanobrevibacter* sp. [12, 13] in particular has been highlighted. Previous studies have indicated a competition for metabolic H_2_ uptake between the methanogenic Archaea and acetogenic bacteria in the animal digestive tract [9, 14, 15]. Reductive acetogenesis is the synthesis of acetate from carbon dioxide and hydrogen and occurs primarily in young rabbits. With age, reductive acetogenesis is partly replaced by methanogenesis [16].

Differences in digestive strategies between the two presented species suggest that microbial populations in the caecum could present dissimilar activity, and these distinctions could affect methanogenesis and caecal fermentation patterns. Although many studies are concerned with caecal fermentation in rabbits, studies of rabbit caecal methanogenesis are limited and have been performed primarily via *in vitro* assays [16, 17, 18, 19]; however, some have been carried out using *in vivo* methods [9, 20].

The objectives of this study were to characterise the brown hare caecal methanogenesis and fermentation pattern and compare the results with those of rabbits by analysing microbial fermentation products such as methane, SCFA and ammonia. Little information is available concerning hare caecal fermentation. Marounek et al. [21] found very low methanogenesis in the hare caecum and a lower total SCFA concentration as well as different proportions of propionate and butyrate compared with the rabbit. In the present study, a comparison between hare and rabbit caecal fermentation was performed, both with and without the addition of substrate, to determine the substrate’s effects and verify the hypothesis that the effect of a shortage of fermentable substrate could explain the differences between these species. Moreover, a comparison of hare and rabbit fermentation patterns could extend our understanding of the digestive physiology of the brown hare. Our results established that the hare caecal methanogenesis still appeared at a very low level despite of substrate addition into inoculums. Additionally, we showed that the hare caecal fermentation pattern differed from that observed in rabbit caecum, particularly by lower total SCFA concentration and higher proportion of propionate which is the main glucogenic SCFA [10].

## Materials and Methods

### Ethics Statement

The experiment was performed after receiving approval from the Local Ethics Commission for Experiments on Animals in Wroclaw, Poland (license no. 2/2009).

### Animals and experimental design

The experiment was performed in two stages which took place in late autumn 2009 and 2010. Fourteen White Giant rabbits (the crossbreed from maternal line Hyla plus 19 and paternal Hyla plus 59) aged 12 weeks were used, seven in the first and seven in the second stage. The animals were purchased from Rabbit Breeding Unit (Koźmin, Poland) and housed in stainless steel cages with free access to drinking water and to the pelleted diet (Table 1) formulated with respect to the rabbit nutritional requirements [22]. After a 3-week adaptation period to the diet, the animals were slaughtered (using xylazine and sodium pentobarbital injections) in order to collect caecal contents for analyses.

**Table 1:** The composition and nutritional values of the basal feed mixture.

**Ingredients^a^**	
Dried grass	130
Dried alfalfa	150
Wheat bran	170
Wheat grain	100
Barley grain	100
Corn	150
Extracted soybean meal	100
Rape meal	50
Rapeseed oil	10
Vitamin-mineral premix^b^	40
**Chemical analysis**	
Crude protein^a^	170
Ether extract^a^	36
Crude fibre^a^	118
Ash^a^	55
Starch^a^	215
Soluble sugars^a^	74
Metabolizable energy (MJ/kg)	10.95

^a^ g/kg

^b^Premix provided per kg of diet: vitamin A, 15000 IU; vitamin D_3,_ 1504.0 IU; vitamin B_1,_ 8.7 mg; vitamin B_2,_ 13.1 mg; vitamin B_6,_ 7.1mg; vitamin B_12_ 0.01 mg; vitamin E, 57.2 mg; vitamin C, 73 mg; vitamin K, 39.9 mg; folic acid, 1.7 mg; niacin, 63.10 mg; pantothenic acid, 30.1 mg; choline 884 mg; inositol, 1334 mg; biotin, 0.272 mg; Mn, 80 mg; Cu, 15 mg; Fe, 197 mg; Zn, 62 mg; Co 0.523 mg; iodine, 0.638 mg; Se, 0.157 mg; F, 0.032 mg.

Fourteen hares were obtained from the Research Centre of Forest Environment and Game Breeding in Zlotowek, Poland. Ten animals were used in the first and four in the second part of the experiment. Hares were kept in the Research Centre in natural conditions, in an area of nine ha containing meadows with a stream, wasteland and woodland, without additional feeding or watering. The stocking rate was 4 animals/ha. The autumn feeding base of hares in Zlotowek comprised the following plant species: *Vaccinium myrtillus* (all plant), *Betula pendula* (shoots and bark), *Prunus padus* (shoots), *Rubus* sp. (all plant), *Viburnum opulus* (shoots), *Rubus idaeus* (all plant), *Alnus glutinosa* (shoots and bark), *Prunus spinosa* (shoots) [23]. The animals were caught using a soft net 400 m long and 150 cm high and placed in wooden cages in which they were transported to a laboratory. Immediately after arrival, the animals were slaughtered (using xylazine and sodium pentobarbital injections) in order to collect caecal contents for analyses.

### *In vitro* caecal fermentation

*In vitro* caecal fermentation of the caecal samples was performed to analyse methane production, SCFA and ammonia concentrations and to measure pH. Methane production and pH were measured in samples from all investigated animals, whereas SCFA and ammonia were analysed only during the second stage of the experiment. Immediately after dissection, the collected caecal contents were mixed with a spatula, and the pH was measured using a pH-meter CP-401 (Elmetron, Zabrze, Poland). Three 20-g subsamples were taken from each animal’s caecal content sample. Each subsample was diluted five-fold with a buffer solution at pH 7.2 [24] and transferred into a 125-ml serum bottle. Two subsamples were prepared for performing the incubation analyses, and the third subsample was not incubated but immediately mixed with HgCl_2_ to stop the fermentation processes (blank sample—B). A total of 1.2 g of wheat bran was added to the first bottle prior to incubation (sample with substrate—S), and the second bottle was incubated without the substrate addition (control sample—C). Both bottles were thoroughly flushed with carbon dioxide to obtain anaerobic conditions and were then hermetically sealed using a manual crimper. The incubation was performed in a shaking water bath at 39°C for 12 h. At the end of incubation, the fermentation gas was sampled with a gas-tight syringe for analyses. Additionally, before the gas analysis, the headspace pressure inside each bottle was measured. In the remaining liquid sample, fermentation was stopped by adding HgCl_2_.

### Analyses

#### Methane

Gas samples were analysed for methane using the Natural Gas Analyser Model Arnel 2008 (Perkin Elmer, Waltham, Massachusetts, USA) with a back-flush system, thermal conductivity detector (TCD) and four columns: two columns packed with DC 230, one column with a 13X molecular sieve and one HST6 column. Helium was used as a carrier gas. Based on the gas pressure and the percentage of methane from the total gas volume, the molar concentration of methane was calculated using the Clapeyron equation.

#### SCFA

Liquid samples were centrifuged at 2,800×g for 20 min and analysed using a gas chromatograph 7890A (Agilent Technologies, Santa Clara, California, USA) equipped with a flame ionisation detector (FID) and an Agilent J&W DB-23 column, with helium as a carrier gas. This analysis was performed to determine the total SCFA and the acetic, propionic, isobutyric, butyric, isovaleric, valeric, isocaproic and caproic acid concentrations.

#### Ammonia and pH

The ammonia in the samples was separated by microdiffusion in Conway units, and the concentration was determined with a Nessler reagent using a Lambda XLS spectrophotometer (Perkin Elmer, Waltham, Massachusetts, USA) at a wavelength of 410 nm. The pH value was measured in all samples.

#### Chemical analyses

In the second stage of the experiment, the aggregate samples of hare and rabbit caecal contents, as well as the sample of wheat bran, were analyzed using standard methodologies [25] for dry matter, crude protein, ether extract, ash, neutral detergent fibre (NDF) and acid detergent fibre (ADF). The gross energy in the samples was measured using an adiabatic oxygen bomb calorimeter (KL-10, Bydgoszcz, Poland).

#### Statistical analyses

Data were subjected to a single factor ANOVA using the Statistica for Windows ver. 8.0 software package (StatSoft, Tulsa, OK, USA) to compare the *in vitro* fermentation parameters of rabbit and hare caecal inoculums. The effect of the species (rabbit versus hare) on the fermentation parameters, the effect of substrate addition to the incubated samples (C versus S) and the effect of the incubation time on C and S sample (B versus C and B versus S) were analysed. Differences between means with *P*<0.05 were accepted as representing statistically significant differences.

## Results and Discussion

The total gas production and concentrations of methane, pH, ammonia and SCFA in the 12-h incubated caecal samples of rabbits and hares are presented in Table 2. The nutrient composition of the hare and rabbit caecal contents, as well as the composition of the substrate are displayed in Table 3.

**Table 2:** The production of gases, ammonia and SCFA of hare and rabbit caecal inocula during 12-hour *in vitro* fermentation.

**Fermentation parameters**		**Hare**			**Rabbit**			**SEM**	***P* value^a^**			
		**B**	**C**	**S**	**B**	**C**	**S**		**species**	**substrate**	**incubation**	
									**H v R**	**C v S**	**B v C**	**B v S**
**Gas production^b^**		-	27.86	74.58	-	57.84	90.96	5.669	0.035	<0.001	-	-
**Methane^b^**		-	0.25	0.22	-	15.49	11.73	1.086	<0.001	0.462	-	-
**pH of fresh content**		5.95	-	-	5.96	-	-	0.065	0.928	-	-	-
**pH of sample**		6.93	6.26	5.93	6.99	6.43	6.08	0.055	0.438	<0.001	<0.001	<0.001
**Ammonia^b^**		3.64	10.75	9.56	2.49	6.58	9.85	0.777	0.306	0.327	<0.001	<0.001
**Total SCFA^b^**		28.4	79.6	120.9	51.8	177.7	246.3	17.47	0.030	0.182	0.002	<0.001
**SCFA** **molar propor-tions^c^**	Acetate	66.6	73.4	72.3	74.5	74.1	70.6	1.377	0.431	0.460	0.517	0.901
	Propionate	11.8	10.4	9.8	8.0	8.0	8.1	0.462	0.004	0.839	0.727	0.515
	Isobutyrate	5.5	3.59	2.35	3.2	2.14	1.98	0.243	0.005	0.080	0.040	0.002
	Butyrate	12.7	9.7	12.6	10.5	12.0	15.2	0.891	0.620	0.188	0.919	0.209
	Isovalerate	2.8	2.00	1.84	2.5	2.40	3.07	0.258	0.425	0.604	0.590	0.967
	Valerate	0.7	0.95	1.05	0.6	1.33	1.16	0.181	0.754	0.884	0.273	0.302
	A:P	5.92	7.6	7.4	9.84	11.5	10.5	0.911	0.056	0.832	0.490	0.636
	P:B	0.96	1.1	0.8	0.85	0.7	0.6	0.056	0.048	0.186	0.930	0.038

B – unincubated (blank) samples, C – samples incubated without the substrate (control), S – samples incubated with substrate (wheat bran) addition

SEM – standard error of the mean

A:P – the ratio of acetate to propionate

P:B – the ratio of propionate to butyrate

^a^Probability of effects of species (H versus R), substrate (C versus S) and incubation time for C samples (B versus C) and for S samples (B versus S)

^b^mmol/kg of caecal content

^c^mol/100 mol of total SCFA concentration

**Table 3:** Chemical composition of hare and rabbit caecal content and of the substrate.

**Parameter**	**Hare caecalcontent**	**Rabbit caecalcontent**	**Wheatbran**
Dry matter (%)	21.68	26.17	91.03
Ash^a^	15.27	7.07	1.97
Crude protein^a^	28.87	35.35	15.16
Crude fibre^a^	18.40	21.25	5.64
Neutral detergent fibre^a^	41.88	51.66	21.29
Acid detergent fibre^a^	23.43	28.28	6.17
Ether extract^a^	1.06	1.53	1.64
Gross energy (MJ/kg)	4.65	5.25	15.20

^a^Percentage of dry matter

### Methane and total gas production

The obtained results show that the volume of gas produced from caecal contents was higher for rabbit samples than for hare samples (*P*<0.05). Moreover, the effect of substrate addition was also noted. Caecal contents incubated with supplementary wheat bran substrate produced higher volumes of fermentation gas than did the samples incubated without substrate (*P*<0.001; Table 2). Although gas production occurred at a lower level in hare caecal fermentation than in rabbit, the addition of substrate increased the gas production in both species, thus indicating an increase in the intensity of microbial fermentation. The volume of produced gas in hare caecal samples incubated with the substrate was even greater than that for rabbit samples incubated without the substrate. In contrast, the addition of the substrate did not affect the production of methane, which was very low in the hare samples, despite the increase in the intensity of microbial fermentation.

The hare caecal samples produced significantly smaller amounts of methane than did the rabbit samples (*P*<0.001; Table 2). During the incubation of rabbit caecal samples, a mean of 13.5 mmol of methane per kg of caecal contents was produced. During hare sample incubation, very little methanogenesis occurred, amounting to approximately 0.2 mmol/kg of methane (Table 2). Hackstein and van Alen [26] reported that methane release by faeces of the blue hare (*Lepus timidus*) was 4 nmol/g.h, on average. Corresponding value in rabbits was 42 nmol/g.h. It is not clear how the measurement of methane release by faeces are relevant for the estimate of methane production *in vivo*. Methane production is influenced by the substrate as shown in rabbits [9], and in ruminants [27]. On the other hand phylogenetic and genetic factors of methane production can not be left aside as shown by differences in animals of the same infraclass, e.g. in vegetarian Marsupialia [26], or even in the same species [28, 29, 30].

The results from the present study confirm the data reported by Marounek et al. [21], who reported very low methane emissions (0.1 mmol/l of headspace gas) in the wild hare caecum. However, the present study was extended to include the substrate (wheat bran) added to caecal inoculums. The presence of substrate greatly increased production of gas and SCFA, both in hare and rabbit caecal cultures, but the production of methane in cultures of hare caecal contents remained low. Because a low level of methane was always released from hare caecal cultures, a lack of methanogens was not responsible for a low level of methane production. In rabbits, the substrate addition did not influence methane production significantly (*P*>0.05), presumably because at low external pH methanogens have only a limited capacity to maintain a constant internal pH [31].

Apart from methanogens and acetogenic bacteria, the third microbial population that consumes hydrogen and exists in animal and human large intestines was also described as sulphate-reducing bacteria that use H_2_ to reduce sulphates and produce sulphides. These bacteria have a higher substrate affinity for H_2_ than methanogenic Archaea [32], but they require sulphate availability for their growth. Caecal pH may determine the pathway of H_2_ disposal because of the different acid sensitivity of these microbial populations. Acetogenic bacteria prefer an acidic pH, sulphate-reducing bacteria prefer alkaline pH, and methanogenic flora favor pH values closer to neutral [32]. The predominance of acetogenesis in rabbits [33] may be caused by the higher acid sensitivity of sulphate-reducing bacteria and methanogens. The replacement of methanogenesis with reductive acetogenesis enhances the production of SCFA per unit of substrate fermented and thus the energy yield for the organism. Belenguer et al. [9] observed higher rabbit caecal methane production through *in vitro* measurements compared with *in vivo* measurements from a respiratory chamber. This finding may be explained by pH values closer to neutrality in the *in vitro* cultures, which seems to be more favourable for Archaea populations. Taken together, our data showed that neither pH, which was similar in our study for rabbit and hare caecal contents, nor availability of fermentable substrates could cause such significant differences in methane formation between rabbits and hares.

Furthermore, acetogenic bacteria are able to grow on substrates other than H_2_/CO_2_, such as monosaccharides [34], which allows them to be more competitive than methanogenic Archaea in digestive tracts characterized by fast passage [35]. This trait may partly explain the lower use of methanogenesis in hares, which have a faster passage rate of digesta compared with rabbits [8]. However, such significant differences in methane production suggest that there may be another reason for this phenomenon.

### pH

As mentioned above, there were no differences between the pH of hare and rabbit samples, neither in the fresh caecal content immediately after squeezing out nor during incubation (Table 2). Incubation with the substrate led to a larger drop in caecal reaction than incubation of control samples for both species, as demonstrated by highly significant differences (*P*<0.001; Table 2).

### Ammonia

From a chemical point of view, caecal pH would be expected to be highly correlated with the main sources of H^+^ and OH^−^, SCFA and ammonia [36]. In the present study, no significant differences in ammonia concentrations in rabbit and hare caecum were found. However, for the samples incubated without substrate, there was a slight tendency for higher ammonia production in hares (10.75 mmol/kg) than in rabbits (6.58 mmol/kg) (Table 2) but the addition of substrate eliminated this difference. Higher ammonia concentration in hare’s than in rabbit’s caecal cultures without substrate probably reflects lower utilization of ammonia for microbial proteosynthesis, which is energy-demanding, thus lower in cultures short of fermentable substrate. In the rumen fluid energy (ATP) production is correlated with SCFA production [37], which was higher in our experiment in rabbit’s than in hare’s cultures. According to Fraga [38], the caecal ammonia concentrations in rabbits fed balanced diets were in the range of 4.5–6 mmol/l, which seems adequate for the appropriate protein microbial synthesis compared with ammonia concentrations in the rumen. Bellier et al. [39] provided similar results for the ammonia concentrations in adult rabbit caecum (6.1 mmol/kg), whereas according to the data presented by Garcia et al. [40], the rabbit caecal ammonia concentration ranged from 1.86 to 23.9 mmol/l.

### SCFA

During microbial digestion of plant cells in herbivorous animals, SCFA are produced primarily as a result of carbohydrate degradation from sources such as hemicellulose, pectin, starch, cellulose, other glucans, and soluble carbohydrates as well as through the bacterial decomposition of proteins. Branched-chain fatty acids, such as isobutyric and isovaleric acids, may be formed as a result of amino acid deamination. In the caecum of both animal species, acetate was the main SCFA, followed by butyrate and propionate.

In the present study, our analyses showed that the total SCFA concentrations in the hare caecal content were lower than the concentrations in the caecal content of rabbits (P<0.05; Table 2). Our data obtained for rabbits are similar to the average values presented by Garcia et al. [40], who reported a mean of 57.4 mmol/l. As expected, the addition of a substrate increased the total SCFA level in the caecal cultures of both investigated species. The lower SCFA production observed in hare than in rabbit caecum is a consequence of differences in digestive physiology and in particular to the poorer digestibility of hemicelluloses in the hare [8] which would lead to lower amounts of fermentative products. In rabbit caecum, acetate is the most abundant SCFA (60–80%), followed by butyrate (8–20%) and propionate (3–10%), resulting in a propionate/butyrate ratio that is less than one [1, 39]. In the present study, similar results were obtained for rabbits, whereas in the hare caecum, this proportion was closer to one and showed a significant increase compared with the rabbit (P<0.05) (Table 2). These differences result from the significantly higher proportions of propionate in the total SCFA concentration in hare than in rabbit caecal contents (P<0.01) (Table 2). The concentration of butyrate in the rabbit caecum was always greater than that of propionate, which represents a divergence from the fermentation ratios found in the caecum of coypus [18] and chickens [41].

The high propionate production may be caused by the hare’s chosen diet of plant parts that are low in fibre and rich in digestible energy [42, 43]. Eating plants low in fibre and rich in energy not only increases energy assimilation but may also lower the hare’s predation risk by enabling the animals to reduce both their foraging activity and the weight of food required for nutrition [44]. There are studies reporting the effects of fat addition on the fermentation profile that have shown an increase in propionate percentage in caecal SCFA in rabbits [45, 46]. However, differences in the bacterial populations that inhabit hare and rabbit caeca more likely contribute to the differences in molar profile of SCFA.

After absorption, propionate is a very effective substrate for glucose synthesis and takes part in glycogenesis and the formation of long-chain fatty acids in the liver. Acetate participates in lipogenesis, milk fat synthesis, cholesterogenesis and ketogenesis, whereas butyrate is used as a source of energy in epithelial cells of the large intestine [10, 47]. The concentration of bacterial fermentation products in the caecum, particularly ammonia and SCFA, reflect the activity of local microflora that determine the course of the processes occurring in the gastrointestinal tract [48]. In addition to propionate, higher isobutyrate production in hares was observed compared with rabbits (*P*<0.01; Table 2). Iso-fatty acids originate from the catabolism of the amino acids by intestinal microflora, so the presence of these acids confirms the occurrence of proteolytic and deamination activity. Isobutyrate is the reduced carbon skeleton of the branched-chain amino acid valine [49]. The diet of wild rabbits is considered to be more abundant in protein than that of hares because wild rabbits primarily choose plants that grow close to their burrows and because this intense grazing in the same area results in the consumption of young, protein-rich plants. In contrast, hares feed over a far larger area and their diet is more diverse [50] but contains less protein and more fibre overall [7]. The higher caecal concentration of iso-acids and the tendency toward higher ammonia level suggest a higher proteolytic and deamination activity in hare caecum compared with that of the rabbit, but this needs to be confirmed by additional research.

### Caecal composition

As shown in Table 3, the hare caecal content is characterized by a higher level of ash and lower levels of crude protein, crude fibre, NDF and ADF compared with the caecal content of the rabbit. However, the higher ash percentage in hares may be caused by the consumption of some soil and sand. These differences in the caecal content between the species is the result of differences in the animals’ diets; the rabbits were fed with standard feed, whereas the hares ate plant food, although the space in which the hares were foraging was sufficient to ensure their food base. Considering the stocking rate and the sufficient access to plant food, these results suggest that the hares were provided with basic nutrients in their diet.

## Conclusion

Hare caecal microflora produce less methane and a lower total SCFA concentration compared with the rabbit. Because the caecal methane production of hares appears to be very low in the presence of the supplementary wheat bran substrate, we may hypothesise that this depends more on local microflora than on access to fodder. Moreover, the caecal fermentation pattern of hares is characterised by higher propionate and isobutyrate levels than observed in rabbit caecum. This could be a result of the slightly different diets and digestive physiologies of these two species; however, these results also suggest differences in the microbial populations that inhabit their caeca. Thus, to explain these questions, further research is required on the hare caecal microflora and their activity. Because the addition of the substrate made some of the parameters in the hare caecum approach those in the rabbit caecum without affecting methanogenesis, further studies on hares and rabbits fed the same diet could elucidate the impact of food on the differences between these species.
